# Passive Exercise of the Hind Limbs after Complete Thoracic Transection of the Spinal Cord Promotes Cortical Reorganization

**DOI:** 10.1371/journal.pone.0054350

**Published:** 2013-01-22

**Authors:** Alessandro Graziano, Guglielmo Foffani, Eric B. Knudsen, Jed Shumsky, Karen A. Moxon

**Affiliations:** 1 School of Biomedical Engineering, Science and Health Systems, Drexel University, Philadelphia, Pennsylvania, United States of America; 2 Department of Physiology and Pharmacology, Drexel University College of Medicine, Philadelphia, Pennsylvania, United States of America; 3 Department of Neurobiology and Anatomy, Drexel University College of Medicine, Philadelphia, Pennsylvania, United States of America; 4 Hospital Nacional de Parapléjicos, Servicio de Salud de Castilla-La Mancha, Toledo, Spain; University of North Dakota, United States of America

## Abstract

Physical exercise promotes neural plasticity in the brain of healthy subjects and modulates pathophysiological neural plasticity after sensorimotor loss, but the mechanisms of this action are not fully understood. After spinal cord injury, cortical reorganization can be maximized by exercising the non-affected body or the residual functions of the affected body. However, exercise per se also produces systemic changes – such as increased cardiovascular fitness, improved circulation and neuroendocrine changes – that have a great impact on brain function and plasticity. It is therefore possible that passive exercise therapies typically applied below the level of the lesion in patients with spinal cord injury could put the brain in a more plastic state and promote cortical reorganization. To directly test this hypothesis, we applied passive hindlimb bike exercise after complete thoracic transection of the spinal cord in adult rats. Using western blot analysis, we found that the level of proteins associated with plasticity – specifically ADCY1 and BDNF – increased in the somatosensory cortex of transected animals that received passive bike exercise compared to transected animals that received sham exercise. Using electrophysiological techniques, we then verified that neurons in the deafferented hindlimb cortex increased their responsiveness to tactile stimuli delivered to the forelimb in transected animals that received passive bike exercise compared to transected animals that received sham exercise. Passive exercise below the level of the lesion, therefore, promotes cortical reorganization after spinal cord injury, uncovering a brain-body interaction that does not rely on intact sensorimotor pathways connecting the exercised body parts and the brain.

## Introduction

Limb amputation, damage to peripheral nerves and spinal cord injury can alter the somatotopic organization of the primary somatosensory cortex [1], [2], [3], [4], [5], [6]. Cortical reorganization subserves functional recovery [7], [8], [9], [10], [11], [12] but excessive reorganization can have pathological consequences, such as phantom limb sensations [13], [14] and neuropathic pain [15], [16], [17], [18]. It is therefore important to fully understand the mechanisms of cortical reorganization and to develop optimal strategies to modulate it after deafferentation [19].

Cortical reorganization after somatosensory deafferentation can lead to the enlargement of cortical representations of intact body areas into cortical representations of deafferented body areas. A conceptually similar cortical enlargement is induced by increasing the activity of a cortical area through enhanced sensory experience [20]. A natural strategy to maximize cortical reorganization after somatosensory deafferentation is therefore to increase the activity of the intact cortex by exercising the non-affected body or the residual functions of the affected body, as investigated in previous works in rat models [9], [21], [22] and in patients with spinal cord injury [23], [24], [25], [26]. An alternative strategy would be to put the overall cortex in a more plastic state, e.g. using systemic drugs or other manipulations that generically promote cortical plasticity [27], [28], [29].

A possible intriguing way to generically promote cortical plasticity is indirectly suggested by exercise therapies employed after spinal cord injury, which are often applied to the *affected* body in order to improve functions below the level of the lesion [30]. For example, passive exercise of the lower limbs after spinal cord injury has been shown to reduce spasticity [31], [32], [33], [34], reduce the rate of bone density loss [35], [36] and reduce lower limb blood pooling [37]. Interestingly, passive exercise also produces the systemic effects that are typical of exercise per se, such as increases in cardiovascular fitness [38], improved circulation [39] and neuroendocrine changes [40]. These systemic effects of exercise are known to have a great impact on brain function [41], as they contribute to stimulate neurogenesis and to increase the levels of growth factors such as BDNF, thereby promoting brain plasticity [42], [43], [44], [45]. Even though at first glance it might appear counter-intuitive, it is reasonable to suggest that exercise below the level of the lesion after complete spinal cord injury might promote cortical reorganization.

**Figure 1:**
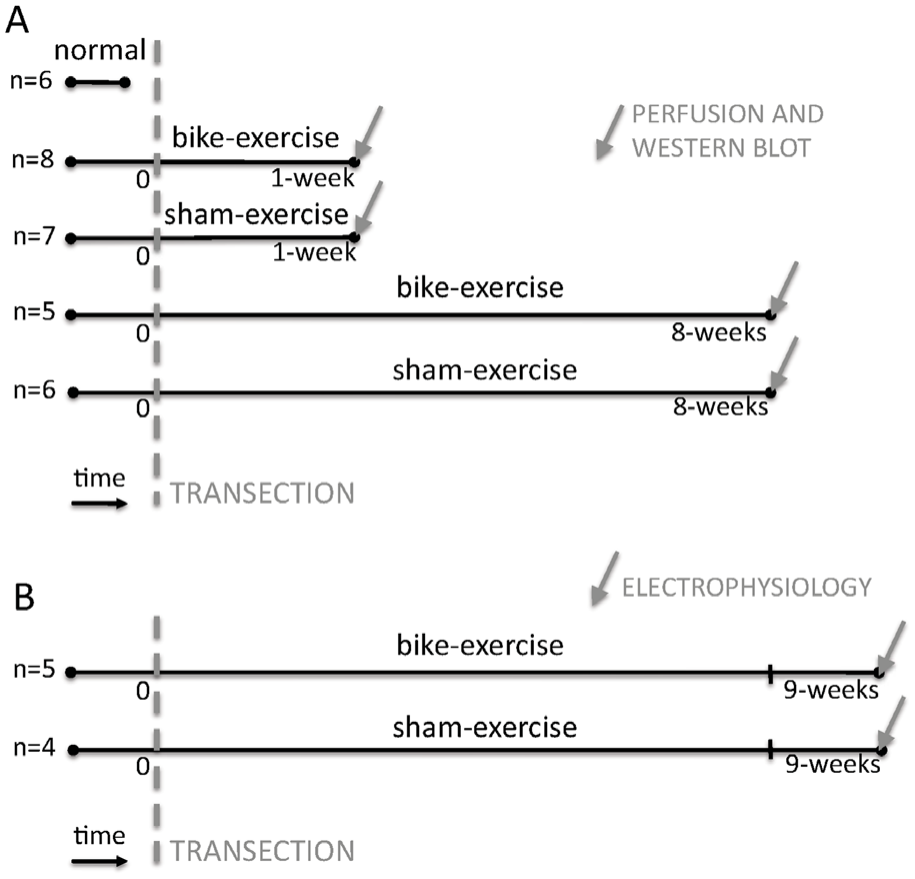
Experimental protocol. (**A**) In 32 rats western blot analysis was performed to assess the levels of plasticity-related proteins in the somatosensory cortex: one group of normal animals (n = 6); two groups of ‘bike-exercise’ transected animals, which received either 1 week (n = 8) or 8 weeks (n = 5) of hindlimb bike exercise after complete thoracic (T9/T10) transection of the spinal cord; two groups of ‘sham-exercise’ transected animals, which received either 1 week (n = 7) or 8 weeks (n = 6) of sham bike exercise after the spinal cord transection. (**B**) In 9 additional rats, an electrophysiological study was performed to assess the functional reorganization of the deafferented somatosensory cortex: one group of ‘bike-exercise’ transected animals (n = 5) and one group of ‘sham-exercise’ transected animals (n = 4). Both groups received real or sham passive hindlimb bike exercise for 8 weeks after transection, and the electrophysiological study was performed one week after the last bike session (i.e. 9 weeks after the spinal transection).

In order to directly test this hypothesis, we applied passive hindlimb bike exercise after complete thoracic transection of the spinal cord in adult rats. The rationale for employing the thoracic transection rat model of spinal cord injury is that it has been shown to produce cortical reorganization associated with alterations of BDNF regulation in the brain [46], [47]. The rationale for employing passive hindlimb bike exercise is three-fold: (*1*) it is well established in rat models of spinal cord injury [48], (*2*) it has direct translational relevance for spinal cord injury patients, and (*3*) it specifically confines the exercise below the level of the lesion. We first investigated whether passive hindlimb bike training after thoracic transection of the spinal cord affected the expression in the somatosensory cortex of proteins associated with plasticity (Fig. 1A). We then verified that these changes in markers of cortical plasticity were associated with increased neurophysiological reorganization of the deafferented hindlimb cortex (Fig. 1B).

## Results

### Hindlimb Bike Exercise after Thoracic Transection of the Spinal Cord Induces Changes in the Levels of Proteins Associated with Plasticity in the Somatosensory Cortex

We performed western blot analysis to assess the levels of plasticity-related proteins in the somatosensory cortex of 32 rats, divided in 5 groups (Fig. 1A): one group of normal animals (n = 6); two groups of ‘bike-exercise’ transected animals, which received either 1 week (n = 8) or 8 weeks (n = 5) of hindlimb bike exercise after complete thoracic (T9/T10) transection of the spinal cord; two groups of ‘sham-exercise’ transected animals, which received either 1 week (n = 7) or 8 weeks (n = 6) of sham bike exercise after the spinal cord transection. Passive hindlimb bike exercise or sham bike exercise started the week after the spinal transection (two 30-min sessions per day on Monday, Wednesday and Friday) and animals were sacrificed within one hour after the last session. This exercise protocol is based on previous work showing that at least 1 week of hindlimb bike exercise after spinal cord injury is necessary to induce significant plastic changes in the spinal cord below the level of the lesion (Keeler et al., 2009). We focused on the following 6 proteins known to be involved in neuronal plasticity in the brain: ADCY1, BDNF and its receptor trkB, GAP43, LINGO-1 and p35 [49], [50], [51], [52], [53], [54], [55], [56], [57], [58], [59], [60], [61], [62]. Data from ‘bike-exercise’ and ‘sham-exercise’ animals were expressed as percentage of normal animals (as in [46]).

**Figure 2:**
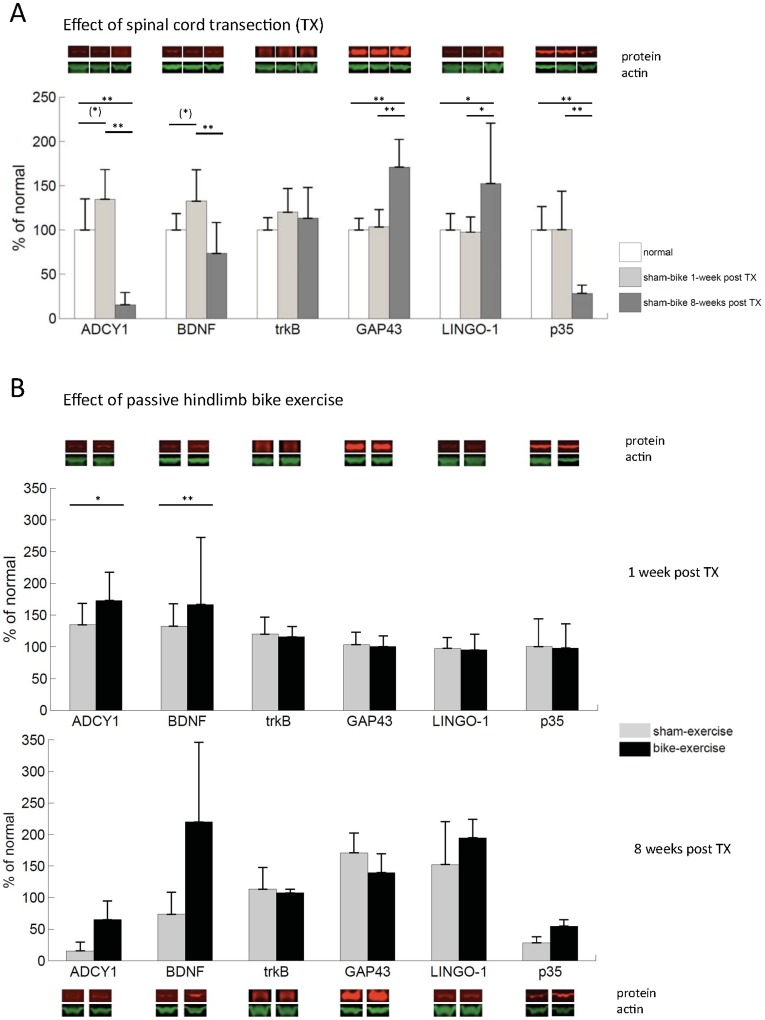
Changes in the levels of proteins associated with plasticity in the somatosensory cortex. (**A**) Effect of spinal cord transection alone (‘sham-exercise’ transected animals compared to normal animals). (**B**) Effect of passive hindlimb exercise. Representative western blot bands are shown for each case. Error bars indicate standard deviations. (*) = p<0.1, * = p<0.05, ** = p<0.01.

It has been previously shown that complete transection of the spinal cord changes the transcriptional activities of several genes related to plasticity in the primary somatosensory cortex, including BDNF, the Nogo receptor and its co-receptor LINGO-1, as assessed by *in situ* hybridization [46]. In order to corroborate the sensitivity of our experimental approach to detect changes in cortical plasticity after spinal cord transection, we first compared ‘sham-exercise’ transected animals to normal animals (Fig. 2A). Spinal transection affected the levels of most of the proteins of interest (two-way ANOVA, time from lesion: F = 3.6, p = 0.0514; interaction time from lesion × protein: F = 12.1, p<0.0001; follow-up one-way ANOVAs, ADCY1: F = 27.4, p<0.0001; BDNF: F = 5.9, p = 0.0120; trkB: F = 0.93, p = 0.41; GAP43: F = 19.5, p<0.0001; LINGO-1: F = 3.6, p = 0.0524; p35: F = 11.2, p = 0.0009). Specifically, 1 week after spinal transection there was only a marginal increase in the level of ADCY1 (post-hoc: p = 0.0505) and BDNF (p = 0.0780), but 8 weeks after spinal transection all proteins but trkB were altered compared to 1 week: ADCY1, BDNF and p35 were decreased (p<0.0001, p = 0.0035, p = 0.0007), whereas GAP43 and LINGO-1 were increased (p<0.0001, p = 0.0279). ADCY1, GAP43, LINGO-1 and p35 were also significantly different 8 weeks after spinal transection compared to normal animals (p = 0.0001, p<0.0001, p = 0.0410, p = 0.0010). These data show that spinal cord injury per se affects the levels of plasticity-related proteins in the rat primary somatosensory cortex.

We then investigated the effect of passive hindlimb bike exercise after spinal transection by jointly analyzing the data from ‘bike-exercise’ and ‘sham-exercise’ animals (Fig. 2B). Passive hindlimb bike exercise after spinal transection increased the levels of plasticity-related proteins in the somatosensory cortex of our animals (three-way ANOVA, exercise: F = 8.7, p = 0.0076). This increase tended to be stronger in animals that received 8 weeks compared to 1 week of bike exercise after spinal cord transection (interaction exercise × weeks: F = 3.2, p = 0.0868), and was highly protein-dependent (interaction exercise × protein: F = 5.1, p = 0.0003). More specifically, the proteins that appeared more sensitive to hindlimb bike exercise were ADCY1 (follow-up two-way ANOVA, exercise: F = 10.7, p = 0.0034) and BDNF (F = 7.2, p = 0.0141). These results show that exercise below the lesion induces changes in the levels of proteins associated with plasticity in the somatosensory cortex after complete spinal cord injury.

### Hindlimb Bike Exercise after Thoracic Transection of the Spinal Cord Enhances the Neurophysiological Reorganization of the Deafferented Hindlimb Cortex

**Figure 3:**
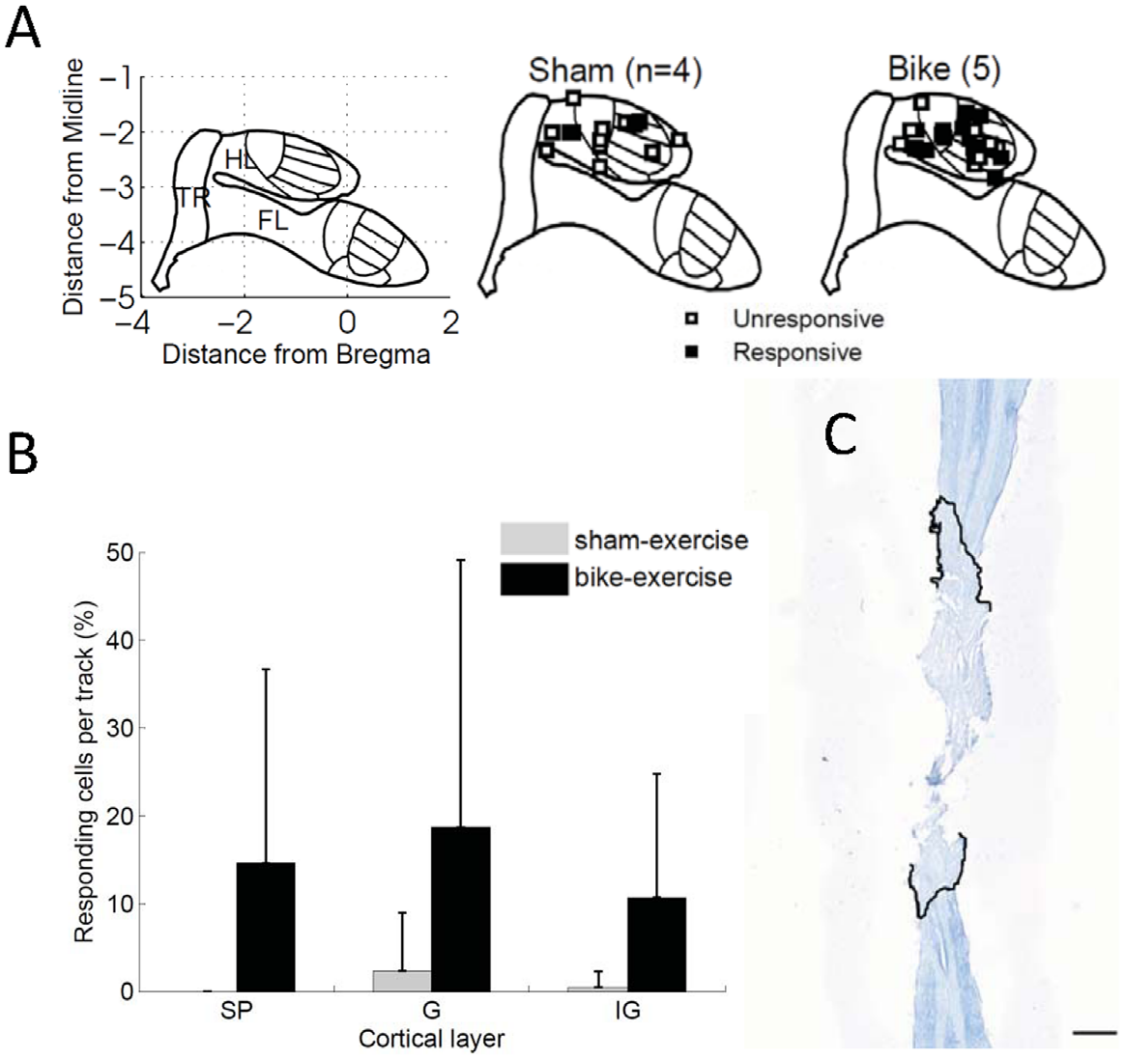
Neurophysiological reorganization of the somatosensory cortex. (A) Cartoon of the hindlimb (HL), forelimb (FL) and trunk (TR) areas in the primary somatosensory cortex in stereotaxic coordinates (left), and visual representation of responsive (filled squares) and unresponsive (empty squares) tracks in the electrophysiological study for sham-exercise transected animals (center) and bike-exercise transected animals (right). (**B**) Percentage of responding cells per track in sham exercise transected animals and bike-exercise transected animals, separated by cortical layer. supragranular (SP), granular (G) and infragranular (IG). In bike-exercise transected animals, the percentage of cells stereotaxically located in the deafferented hindlimb cortex that responded to stimulation of the intact forelimb dramatically increased (two-way mixed ANOVA, exercise: F = 7.54, p = 0.0091) at all cortical layers (interaction exercise × cortical layer: F = 0.88, p = 0.42). Error bars indicate standard deviations. (**C**) Nissl/myelin staining of the spinal cord of a representative animal that received bike exercise after spinal cord transection. No cell bodies or axons were observed in the transection site (marked by the black lines). The extent of this representative injury was comparable with the other injuries. Scale bar: 1.0 mm.

In order to verify the functional relevance of the above changes in proteins associated with cortical plasticity, we directly assessed whether passive hindlimb bike exercise after spinal cord transection affected the neurophysiological reorganization of the deafferented hindlimb cortex. To this end, we studied two additional groups of ‘bike-exercise’ transected animals (n = 5) and ‘sham-exercise’ transected animals (n = 4) (Fig. 1B). These new groups received passive hindlimb real or sham bike exercise for 8 weeks after transection. One week after the last bike session (i.e. 9 weeks after the spinal transection), animals were anesthetized to perform an acute, single-neuron mapping study in the deafferented hindlimb cortex. Similar to our previous study [9], high-impedance single tungsten microelectrodes were inserted into the hindlimb primary somatosensory cortex with multiple penetrations (4–6 per animal), and single neurons from the supragranular (bike: n = 142; sham: n = 64) granular (bike: n = 95; sham: n = 65) and infragranular (bike: n = 253; sham: n = 121) layers were isolated and identified as either responsive or not responsive to cutaneous stimulation of the forelimbs (Fig. 3A, Table 1).

**Table 1:** Electrophysiological data.

	Responsive tracks per animal				Responsive cells per animal			
	SG	G	IG	TOT	SG	G	IG	TOT
SHAM 1	0 of 4	0 of 4	0 of 4	0 of 4	0 of 13	0 of 20	0 of 30	0 of 63
SHAM 2	0 of 5	0 of 5	0 of 5	0 of 5	0 of 14	0 of 18	0 of 18	0 of 50
SHAM 3	0 of 4	0 of 4	0 of 4	0 of 4	0 of 16	0 of 7	0 of 28	0 of 51
SHAM 4	0 of 4	2 of 4	1 of 4	2 of 4	0 of 17	2 of 17	1 of 35	3 of 69
TOT	**0 of 17**	**2 of 17**	**1 of 17**	**2 of 17**	**0 of 60**	**2 of 62**	**1 of 111**	**3 of 233**
BIKE 1	6 of 6	6 of 6	5 of 6	6 of 6	13 of 54	10 of 30	12 of 54	35 of 138
BIKE 2	1 of 5	1 of 5	2 of 5	3 of 5	1 of 22	1 of 18	3 of 55	5 of 95
BIKE 3	1 of 4	1 of 4	4 of 4	4 of 4	1 of 12	1 of 7	4 of 56	6 of 75
BIKE 4	2 of 5	2 of 5	2 of 5	2 of 5	6 of 30	7 of 22	8 of 48	21 of 100
BIKE 5	0 of 4	0 of 4	0 of 4	0 of 4	0 of 24	0 of 18	0 of 40	0 of 82
TOT	**10 of 24**	**10 of 24**	**13 of 24**	**15 of 24**	**21 of 142**	**19 of 95**	**27 of 253**	**57 of 490**

SG = supragranular, G = granular, IG = infragranular.

In sham-exercise transected animals, the percentage of cells stereotaxically located in the deafferented hindlimb cortex that responded to stimulation of the intact forelimb was extremely low: 1.3% considering all cells (n = 233), 0% in the supragranular layers, 3.2% in the granular layers, and <1% in the infragranular layers. In bike-exercise transected animals, the percentage of cells stereotaxically located in the deafferented hindlimb cortex that responded to stimulation of the intact forelimb dramatically increased at all cortical layers: 11.6% considering all cells (n = 490, two-proportion test: p<0.0001), 14.8% in the supragranular layers (p = 0.0019), 20% in the granular layers (p = 0.0030) and 10.7% in the infragranular layers (p = 0.0014). Essentially the same results were obtained with a two-way ANOVA, considering each track as an independent sample (Fig. 3B). The data for each individual animal are given in Table 1. The completeness of the spinal cord transection was histologically verified in all animals at the end of the study (Fig. 3C). There were no differences (t-test, p = 0.53) in the rostrocaudal extent of the lesion between animals that received bike exercise (6.2±0.6 mm) and animals that received sham exercise (6.0±0.4 mm) after the spinal transection.

These results represent the electrophysiological counterpart to the exercise-induced protein regulation reported in the previous section and overall show that exercise below the level of the lesion promotes cortical reorganization after complete spinal cord injury.

## Discussion

We previously showed that exercise (treadmill) above the level of the lesion promotes cortical reorganization in adult rats spinalized as neonates [9], [22]. The main result of the present work is that passive exercise *below* the level of the lesion promotes cortical reorganization in adult rats spinalized as adults. Specifically, we show that passive hindlimb bike exercise after thoracic transection of the spinal cord induces changes in the levels of proteins associated with plasticity in the sensorimotor cortex and enhances the neurophysiological reorganization of the deafferented hindlimb cortex.

### Hindlimb Bike Exercise after Thoracic Transection of the Spinal Cord Induces Changes in the Levels of Proteins Associated with Plasticity in the Sensorimotor Cortex

Spinal cord transection alone (‘sham-exercise’ transected animals compared to normal animals) had a profound impact on the levels of plasticity-related proteins in the somatosensory cortex. On the one hand, ADCY1 and BDNF tended to increase 1 week after spinal transection and significantly decreased 8 weeks after spinal transection – even below normal levels for ADCY1, together with p35. On the other hand, GAP43 and LINGO-1 increased 8 weeks after the spinal transection.

BDNF is critical for neuronal growth and differentiation (Huang and Reichardt, 2001) and for the establishment of long-term synaptic plasticity [55], [56], whereas LINGO-1 promotes neuronal survival and branching [54], [58] but negatively regulates central myelination and axonal sprouting after spinal cord injury [52], [53]. These two proteins have been previously implicated in brain reorganization after spinal cord injury, with complex temporal patterns of up-regulation and down-regulation [46], [47], [63], [64]. In the only previous study specifically investigating the neocortex, Endo et al. [46] used in situ hybridization to quantify changes in the levels of BDNF and LINGO-1 mRNAs after spinal cord transection, reporting changes in gene expression that only in part match the changes in protein levels we observed.

After spinal cord transection in the absence of passive exercise, LINGO-1 mRNA was reduced in the deafferented hindlimb cortex and the adjacent forelimb cortex [46], while in our study protein levels are unchanged 1 week after transection and are even increased 8 weeks after transection. Endo et al. measured mRNA levels in discrete cortical sub-regions and the observed changes involved only a subset of these regions. Because we extracted total protein from the whole sensorimotor cortex, it is possible that changes affecting only part of it where diluted and hence not detectable. A more intriguing possibility, however, is that posttranscriptional and posttranslational modifications are responsible for the uncoupling of LINGO-1 mRNA and protein levels. One important mechanism is represented by micro-RNAs (miRNA), which affect protein translation by binding to their target mRNA and effectively controlling protein levels (see for example [65]). Moreover, LINGO-1 proteins form very stable membrane-bound homotetramers that act as a scaffold with multiple sites available for protein-protein interactions [66]. It is therefore likely that numerous post-translational modifications may affect its subcellular targeting, processing and degradation rate, hence affecting the net protein level, at least in part independent of its mRNA levels. Since ours is the first study to address changes in cortical LINGO-1 protein levels after spinal cord transection, such possibilities cannot be ruled out and suggest interesting new lines of investigation on the mechanisms of cortical reorganization after spinal cord injury and new therapeutic options.

Similar considerations hold for the difference between mRNA and protein levels of BDNF. Endo et al. [46] showed long-lasting up-regulation of BDNF mRNA levels in transected rats; on the contrary, 8 weeks after transection we observed increased BDNF levels only in transected animals under bike exercise regimen, while in non-exercising transected animals BDNF levels were actually decreased. Again, it is possible that post-transcriptional and post-translational mechanisms uncoupled the regulation of protein levels from mRNA levels, causing the discrepancy between the two studies. Our study further shows that other proteins known to be associated with different aspects of morphological and functional plasticity are regulated after spinal cord transection. These include ADCY1, which is involved in long-term synaptic plasticity, learning and memory [51]; GAP43, which is critical for the normal establishment of ordered topography in the developing barrel cortex [67], [68] and mediates functional recovery after lesions in the adult central nervous system [21], [57], [60]; p35, which is regulated in neurodegeneration [61], has an important neuroprotective role in vivo [49] and contributes to different forms of synaptic plasticity [62]. A complex network of proteins is therefore involved in cortical reorganization after spinal cord injury.

Passive hindlimb bike exercise consistently modulated only the levels of two of the above proteins (increasing them): BDNF and ADCY1. On the one hand, exercise has been associated with increased brain levels of BDNF in several previous studies [45], [69], [70], [71]. Our data suggest that exercise-dependent modulation of BDNF levels in the brain is not necessarily mediated by the sensorimotor system, but could also be mediated by cardiovascular and/or neuroendocrine mechanisms [41]. On the other hand, the involvement of ADCY1 is intriguing because of the critical role of this protein in multiple forms of plasticity, including the formation of cortical barrels [72], [73], [74], experience-dependent strengthening of whisker thalamic relay synapses [51] and the refinement of retinal projection maps [75], [76] during development. It therefore seems possible that molecular mechanisms mediating cortical plasticity during development might be reactivated in adulthood after a massive injury such as spinal cord injury. The demonstration that cortical ADCY1 has a causal role in central sensitization and behavioral allodynia after peripheral nerve injury [77] and, more recently, that it affects behavioral sensitization by controlling PKMz levels independently of transcription [78] has important clinical implications, as its regulation may represent the mechanism underlying the emergence and maintenance of pain after spinal cord injury. Our data indicate that passive exercise, even in absence of sensorimotor spinal connections, can modulate these molecular mechanisms and suggest possible novel targets to selectively control central pain after spinal cord injury.

### Hindlimb Bike Exercise after Thoracic Transection of the Spinal Cord Enhances the Neurophysiological Reorganization of the Deafferented Hindlimb Cortex

The altered levels of plasticity-related proteins were not sufficient – without passive bike exercise – to induce a detectable electrophysiological reorganization: in ‘sham-exercise’ transected animals, the probability of neurons in the deafferented hindpaw cortex to respond to forepaw/forelimb tactile stimulation 8 weeks after spinal transection was extremely low, as expected from normal animals under deep anesthesia [9]. This observation is consistent with the absence of cortical reorganization reported by Jain et al. (1995) based on the responses to tactile stimuli after dorsal columns section [79], and is not in contradiction with the reorganization observed with functional magnetic resonance imaging (fMRI), based on the responses to high-intensity electrical stimuli after complete transection of the spinal cord [46], [80]. In fact, a similar dependence on stimulus-intensity is observed immediately after spinal cord injury [81], when the responses to low-intensity electrical stimuli are not sufficiently consistent to uncover cortical reorganization, but cortical reorganization is revealed by high-intensity stimuli. The dependence of long-term cortical reorganization after spinal cord injury on stimulus-intensity, possibly due to differences between dorsal columns inputs and spinothalamic inputs [82], is beyond the scope of the present study and will deserve further investigation.

The data presented here show that passive bike training after spinal cord transection is indeed sufficient to increase the neural responsiveness of the deafferented hindpaw cortex to tactile stimuli delivered to the forepaw/forelimb under deep anesthesia. This increase in responsiveness was observed at all cortical layers – differently from our previous experiments with forelimb training in neonatally spinalized rats, in which we did not observe changes in the granular layer [9]. Interestingly, a similar increase in responsiveness of the hindpaw cortex to tactile stimuli delivered to the forepaw/forelimb can be obtained by decreasing the depth of anesthesia [83]. This suggests that the exercise-dependent increase of neural responsiveness might be mediated not only by axonal sprouting [84], [85], [86], but also by the alteration of the dynamic equilibrium of dendritic spines and axonal boutons between interneurons and pyramidal neurons [10], [87], possibly as a compensatory process to maintain the delicate cortical balance between excitation and inhibition [88], or even by the alteration of more subtle intrinsic properties of cortical neurons. Combining our electrophysiological and molecular observations, even though we did not show a causal link, it is tempting to suggest that BDNF and ADCY1 could be particularly critical for the reorganization of the cortical architecture dedicated to the processing of dorsal column inputs. Nonetheless, because the neurophysiological reorganization we observed in the deafferented hindlimb cortex was not layer-specific, we cannot exclude a subcortical contribution to the cortical reorganization [12], [89], [90], [91], [92], [93]. We speculatively propose that the cardiovascular and neuroendocrine systemic effects produced by passive exercise can boost or simply accelerate the cascade of plasticity-related events that are already occurring in the massively deafferented somatosensory cortex after spinal cord injury.

Independently of the exact mechanism, our data show that passive exercise of the lower limbs after complete transection of the spinal cord is indeed sufficient to promote cortical reorganization.

### Conclusions

We showed that passive exercise below the level the lesion in rats both alters plasticity-related proteins in the sensorimotor cortex and promotes an electrophysiologically measurable reorganization in the deafferented hindlimb cortex after complete spinal cord injury. These results support the important role of physical exercise for learning and plasticity, suggest that exercise should be considered – among its other effects – as a rehabilitation strategy that generically promotes cortical plasticity, and uncover a brain-body interaction that does not rely on intact sensorimotor pathways connecting the exercised body parts and the brain.

## Materials and Methods

A total of 41 adult Sprague-Dawley were used in this study. The different experimental groups are indicated in Figure 1. In 32 animals we used western blot analysis to assess whether passive hindlimb bike training after thoracic transection of the spinal cord affected the expression in the somatosensory cortex of proteins associated with plasticity (Fig. 1A). In 9 additional animals we performed single-neuron mapping in the deafferented somatosensory cortex at the end of the study to electrophysiologically verify the presence of exercise-dependent cortical reorganization (Fig. 1B). All procedures were performed under the guidelines of the National Institutes of Health, and approved by the Institutional Animal Care and Use Committee of Drexel University.

### Spinal Cord Transection

Animals received a complete thoracic transection of the spinal cord with procedures that are similar to our previous studies [81], [94]. Briefly, adult female Sprague-Dawley (Charles River) rats were anesthetized with isoflurane (2–3% with oxygen) and the spinal cord was exposed by laminectomy at the T8/T9 level. The cord was transected with iridectomy scissors followed by aspiration of tissue within the cavity. A collagen matrix, Vitrogen, was injected into the site of the transection to fill the cavity. The muscle and skin were sutured in layers with 5-0 silk. Animals were then warmed, and when they became active, returned to their home cages. Bladders were manually expressed until the animals were able to void on their own. Animals were housed under a 12 h light/dark cycle (lights on at 07∶00) with free access to food and water.

### Passive Hindlimb Bike Exercise

As in previous studies, hindlimb bike exercise consisted of two 30-minute sessions with a 10-minute break, 3 days per week (M, W and F) [48]. This exercise regimen involved suspending the rats on a sling with the hindlimbs hanging down and the hind feet strapped onto the pedals of a bicycle-type device that was driven by a motorized belt. The exercise consisted of a pedaling motion that flexed one limb while extending the other without overstretching the limbs. Cycling speed was 0.5 Hz. This was, therefore, a passive exercise of the hindlimbs only. Animals did not watch the exercised hindlimbs. Sham exercise consisted of placing the animals on the bike for 70 minutes, 3 days per week, but without moving the pedals.

### Western Blot Analysis

Immediately following their last exercise session, animals were anesthetized with isoflurane and then decapitated. The brain and spinal cord were immediately removed, sliced on ice into 2 mm thick coronal slices and flash-frozen on dry ice. We focused on the sensorimotor cortex. Each tissue sample was placed into a FastPrep 120 tube (MP Biomedicals, Irvine, CA) in cold homogenization buffer and homogenized with FastPrep 120 for 40 sec, centrifuged at 1000 g for 10 min, transferred into 1.5 ml microfuge tubes and centrifuged at 20,000 g for 15 min. Protein concentration was assessed by using BioRad DC protein assay method (BioRad, Hercules, CA). Homogenates were fast frozen on dry ice and stored at −80°C until further processing. Proteins were run on pre-cast 26 well SDS gels (BioRad) either 10% or 4–15% gradient, depending upon expected protein molecular weight. Protein standards (Biorad) were used to determine protein size. For each antibody an initial amount of 30 µg total protein/well was loaded at four different dilutions (1∶1, 1∶2, 1∶5, 1∶10). Once the optimal dilution was identified, it was held constant across all samples. Gelled proteins were transferred to 0.2 µ nitrocellulose membrane using I Blot system (Invitrogen). After transfer the membrane was incubated for 1 hr at room temperature in Li Cor western block (Li Cor, Lincoln NE) solution and then overnight at 4°C in primary antibody solution (Li Cor Block +0.1% Tween) with one of the following primary antibodies: adenylate cyclase isoform 1 (ADCY1; Bioworlde BS2391), brain derived neurotrophic factor (BDNF; Santa Cruz SC-20981), tyrosine kinase B (trkB; Millipore 07–225), 43 kD growth associated protein (GAP43; Epitomics 2259-1), leucine-rich repeat and Ig domain-containing Nogo receptor-interacting protein (LINGO-1; Santa Cruz SC-134597), Cyclin-dependent kinase 5 activator 1 (p35; Bioworlde BS2065). Antibody anti Beta-actin (Sigma A5441) was added to the incubation solution for endogenous control. After incubation in primary antibodies the blots were rinsed 3×10 min in PBS +0.1% Tween and incubated 1 hr and RT in block solution containing the following secondary antibodies developed in goat: anti-rabbit IgG IRDye700DX-conjugated, cat #611-130-122 and anti-mouse IgG IRDye800DX-conjugated, cat #610-132-121 (Rockland, Gilbertsville, PA) and HRP conjugated streptactin (BioRad 161-0382), then washed 3×10 min in PBS+Tween and rinsed in PBS. Processed membranes were exposed on Li-Cor Odyssey infrared imager at both 700 and 800 nm wavelengths for appropriate exposure times. All protein level values were obtained on the gel image by subtracting the background intensity from the signal for the protein of interest. The same procedure was performed for the control protein (Beta-actin) and each blot was normalized to its corresponding actin value. For statistical and illustration purposes, all western blot data for each animal and each protein were divided by the average value of the corresponding protein in the normal animals group and expressed as percentage. Note that in this way the average value for normal animals is 100% but the variability between animals (see error bars) is maintained.

### Electrophysiology

Acute single-neuron mapping of the deafferented hindpaw cortex was performed at the end of the study with similar techniques as in our previous study [9]. Rats were anesthetized by intraperitoneal injection of urethane anesthesia (1.3 g/Kg) and placed in a stereotaxic frame. Craniotomies were performed over either the right or left cortex to expose the hindlimb representations in the primary somatosensory cortex. The stereotaxic coordinates for hindlimb craniotomy were from 0 to 3 mm posterior to bregma and from 2 to 3 mm lateral [95]. Electrode penetrations were defined using the stereotaxic coordinates for the hindlimb somatosensory cortex [96], [97]. For all animals, the anesthesia level was maintained at Stage III-4 [98].

A high impedance (10 MΩ) tungsten microelectrode (FHC, Inc, Bowdoin, ME) was mounted on a stereotaxic electrode manipulator. A ground wire was inserted into the brain adjacent to the craniotomies. The microelectrode was then moved to the anterior-posterior and medial-lateral coordinates that defined a predetermined location above the hindlimb somatosensory cortex, and lowered, perpendicular to the surface of the brain, to penetrate the dura and pia. The microelectrode was then slowly inserted into the brain.

The signals from the microelectrode were continuously monitored on the oscilloscope and audio speakers as the electrode was lowered. When a neuron was encountered, the dorsal/ventral coordinates of the cell were noted. Two experimenters then determined whether the identified cell responded to sensory stimulation. The first experimenter, with knowledge of the electrode placement, used wooden probes to touch the hair/skin on the forelimb and shoulder. The second experimenter, blind to the position of the electrode and treatment group of the animal, determined if the cell responded to the stimulus, predominately by listening for a change in firing rate. If the cell did not modulate its firing rate in response to the stimulation, the cell was noted as negative. If the cell did modulate its firing rate, the cell was noted as positive. If the cell was noted as positive, then the receptive field of the cell was identified by tapping locations on the body rostral to the level of the injury. Stimulation of any body surface that modulated the cell’s firing rate was considered part of the cell’s receptive field. To ensure that tapping forces between animals and across sites were uniform, the responses elicited by the wooden probe were periodically compared to responses elicited by von Frey filaments to calibrate the stimulus applied by the wooden probe. The stimulation consisted of pressing a filament gently against the skin, perpendicular to its surface until the filament bent 90 degrees. This procedure was done 5 times for each filament and skin site, to ensure reproducibility of the results. The filament necessary to elicit a response similar to the wooden probe was noted and compared across animals and locations. The filaments required to produce an equivalent response ranged from 4.31 (bending force of 2 g) to 4.93 (bending force of 8 g) across animals and animal groups. There were no identifiable differences in the distribution of filaments used between the groups.

After a cell was characterized, the microelectrode was moved at least 50 microns deeper (with respect to the cortical surface) before another cell could be identified in the same penetration to ensure a new cell was encountered. For every cell identified, the stereotaxic coordinates of the microelectrode position were identified allowing us to evaluate neuronal responsiveness for each layer of the cortex. To minimize tissue damage and its possible effects on cell responsiveness during later penetrations, no more than 6 penetrations were performed per animal.

### Perfusion and Histological Processing of the Brain and Spinal Cord

At the end of the mapping sessions, the rats were perfused transcardially with buffered saline, followed by buffered 2% paraformaldehyde, and then by buffered 2% paraformaldehyde containing 10% sucrose. The cortex was removed and flattened between two glass slides. The tissue was cryoprotected in 30% sucrose and sectioned (70 microns parallel to the pial surface) frozen on a sliding microtome. Series of these sections from the cortex were stained for cytochrome oxidase (CO) activity [99]. Spinal cords were removed and placed in phosphate buffer containing 30% sucrose for 72 h. Specimens were frozen in OCT and sectioned on a freezing microtome at 20 µm. The transection segments of the spinal cords were sectioned parasagitally, and alternate sections were Nissl-myelin stained. The resulting sections were examined under a microscope to confirm completeness of the transection.

### Statistical Analyses

To assess the effects of spinal cord transection alone on plasticity-related proteins in the sensorimotor cortex, we compared ‘sham-exercise’ transected animals to normal animals by entering the data into a two-way independent-measures analysis of variance (ANOVA). The factors were ‘protein’ (ADCY1, BDNF, trkB, GAP43, LINGO-1 and p35), and ‘time from lesion’ (normal, 1 week after transection, 8 weeks after transection). In case of significant interaction, we performed follow-up one-way ANOVAs on individual proteins, followed by Fisher’s post-hoc test (as in Endo et al., 2007).

To assess the effects of passive hindlimb bike exercise after spinal transection on plasticity-related proteins in the sensorimotor cortex, the data from ‘bike-exercise’ and ‘sham-exercise’ animals were entered into a three-way independent-measures analysis of variance (ANOVA). The factors were ‘exercise’ (bike or sham), ‘protein’ (ADCY1, BDNF, trkB, GAP43, LINGO-1 and p35) and ‘weeks’ (1 or 8 weeks). In case of significant interactions, we performed follow-up two-way ANOVAs on individual proteins.

To assess the effects of passive hindlimb bike exercise after spinal transection on the electrophysiological reorganization of the deafferented cortex, we performed two types of analyses. First, the percentage of cells stereotaxically located in the deafferented hindlimb cortex that responded to stimulation of the intact forelimb were compared between bike animals and sham animals using two-proportion tests, separately for all cells and for each cortical layer. Second, the percentage of cells *per track* stereotaxically located in the deafferented hindlimb cortex that responded to stimulation of the intact forelimb were entered into a two-way mixed ANOVA, considering each track as an independent sample. The factors were exercise (bike or sham, independent measures) and cortical layer (supragranular, granular, infragranular, repeated measures).

Results were considered significant at p<0.05.

## References

[1] Wall PD, Egger MD (1971) Formation of new connexions in adult rat brains after partial deafferentation. Nature 232: 542–545. 4328622 10.1038/232542a0

[2] Calford MB, Tweedale R (1988) Immediate and chronic changes in responses of somatosensory cortex in adult flying-fox after digit amputation. Nature 332: 446–448. 3352742 10.1038/332446a0

[3] Pons TP, Garraghty PE, Ommaya AK, Kaas JH, Taub E, et al (1991) Massive cortical reorganization after sensory deafferentation in adult macaques. Science 252: 1857–1860. 1843843 10.1126/science.1843843

[4] Jain N, Catania KC, Kaas JH (1997) Deactivation and reactivation of somatosensory cortex after dorsal spinal cord injury. Nature 386: 495–498. 9087408 10.1038/386495a0

[5] Florence SL, Taub HB, Kaas JH (1998) Large-scale sprouting of cortical connections after peripheral injury in adult macaque monkeys. Science 282: 1117–1121. 9804549 10.1126/science.282.5391.1117

[6] Kaas JH (2000) The reorganization of somatosensory and motor cortex after peripheral nerve or spinal cord injury in primates. Prog Brain Res 128: 173–179. 11105677 10.1016/S0079-6123(00)28015-1

[7] Green JB, Sora E, Bialy Y, Ricamato A, Thatcher RW (1998) Cortical sensorimotor reorganization after spinal cord injury: an electroencephalographic study. Neurology 50: 1115–1121. 9566404 10.1212/wnl.50.4.1115

[8] Nishimura Y, Onoe H, Morichika Y, Perfiliev S, Tsukada H, et al (2007) Time-dependent central compensatory mechanisms of finger dexterity after spinal cord injury. Science 318: 1150–1155. 18006750 10.1126/science.1147243

[9] Kao T, Shumsky JS, Murray M, Moxon KA (2009) Exercise induces cortical plasticity after neonatal spinal cord injury in the rat. J Neurosci 29: 7549–7557. 19515923 10.1523/JNEUROSCI.2474-08.2009 PMC2743445

[10] Ghosh A, Peduzzi S, Snyder M, Schneider R, Starkey M, et al (2011) Heterogeneous spine loss in layer 5 cortical neurons after spinal cord injury. Cereb Cortex 22: 1309–1317. 21840844 10.1093/cercor/bhr191

[11] Ghosh A, Sydekum E, Haiss F, Peduzzi S, Zorner B, et al (2009) Functional and anatomical reorganization of the sensory-motor cortex after incomplete spinal cord injury in adult rats. J Neurosci 29: 12210–12219. 19793979 10.1523/JNEUROSCI.1828-09.2009 PMC6666156

[12] Bowes C, Massey JM, Burish M, Cerkevich CM, Kaas JH (2012) Chondroitinase ABC promotes selective reactivation of somatosensory cortex in squirrel monkeys after a cervical dorsal column lesion. Proc Natl Acad Sci U S A 109: 2595–2600. 22308497 10.1073/pnas.1121604109 PMC3289303

[13] Moore CI, Stern CE, Dunbar C, Kostyk SK, Gehi A, et al (2000) Referred phantom sensations and cortical reorganization after spinal cord injury in humans. Proc Natl Acad Sci U S A 97: 14703–14708. 11114177 10.1073/pnas.250348997 PMC18982

[14] Simoes EL, Bramati I, Rodrigues E, Franzoi A, Moll J, et al (2012) Functional expansion of sensorimotor representation and structural reorganization of callosal connections in lower limb amputees. J Neurosci 32: 3211–3220. 22378892 10.1523/JNEUROSCI.4592-11.2012 PMC6622024

[15] Flor H, Elbert T, Knecht S, Wienbruch C, Pantev C, et al (1995) Phantom-limb pain as a perceptual correlate of cortical reorganization following arm amputation. Nature 375: 482–484. 7777055 10.1038/375482a0

[16] Lotze M, Grodd W, Birbaumer N, Erb M, Huse E, et al (1999) Does use of a myoelectric prosthesis prevent cortical reorganization and phantom limb pain? Nat Neurosci 2: 501–502. 10448212 10.1038/9145

[17] Klit H, Finnerup NB, Jensen TS (2009) Central post-stroke pain: clinical characteristics, pathophysiology, and management. Lancet Neurol 8: 857–868. 19679277 10.1016/S1474-4422(09)70176-0

[18] Wrigley PJ, Press SR, Gustin SM, Macefield VG, Gandevia SC, et al (2009) Neuropathic pain and primary somatosensory cortex reorganization following spinal cord injury. Pain 141: 52–59. 19027233 10.1016/j.pain.2008.10.007

[19] Engineer ND, Riley JR, Seale JD, Vrana WA, Shetake JA, et al (2011) Reversing pathological neural activity using targeted plasticity. Nature 470: 101–104. 21228773 10.1038/nature09656 PMC3295231

[20] Feldman DE, Brecht M (2005) Map plasticity in somatosensory cortex. Science 310: 810–815. 16272113 10.1126/science.1115807

[21] Girgis J, Merrett D, Kirkland S, Metz GA, Verge V, et al (2007) Reaching training in rats with spinal cord injury promotes plasticity and task specific recovery. Brain 130: 2993–3003. 17928316 10.1093/brain/awm245

[22] Kao T, Shumsky JS, Knudsen EB, Murray M, Moxon KA (2011) Functional role of exercise-induced cortical organization of sensorimotor cortex after spinal transection. J Neurophysiol 106: 2662–2674. 21865438 10.1152/jn.01017.2010 PMC3214119

[23] Thomas SL, Gorassini MA (2005) Increases in corticospinal tract function by treadmill training after incomplete spinal cord injury. J Neurophysiol 94: 2844–2855. 16000519 10.1152/jn.00532.2005

[24] Winchester P, McColl R, Querry R, Foreman N, Mosby J, et al (2005) Changes in supraspinal activation patterns following robotic locomotor therapy in motor-incomplete spinal cord injury. Neurorehabil Neural Repair 19: 313–324. 16263963 10.1177/1545968305281515

[25] Hoffman LR, Field-Fote EC (2007) Cortical reorganization following bimanual training and somatosensory stimulation in cervical spinal cord injury: a case report. Phys Ther 87: 208–223. 17213410 10.2522/ptj.20050365

[26] Knikou M (2012) Plasticity of corticospinal neural control after locomotor training in human spinal cord injury. Neural Plast 2012: 254948. 22701805 10.1155/2012/254948 PMC3373155

[27] Juliano SL, Ma W, Eslin D (1991) Cholinergic depletion prevents expansion of topographic maps in somatosensory cortex. Proc Natl Acad Sci U S A 88: 780–784. 1992469 10.1073/pnas.88.3.780 PMC50897

[28] Kilgard MP, Merzenich MM (1998) Cortical map reorganization enabled by nucleus basalis activity. Science 279: 1714–1718. 9497289 10.1126/science.279.5357.1714

[29] Conner JM, Chiba AA, Tuszynski MH (2005) The basal forebrain cholinergic system is essential for cortical plasticity and functional recovery following brain injury. Neuron 46: 173–179. 15848797 10.1016/j.neuron.2005.03.003

[30] Hutchinson KJ, Gomez-Pinilla F, Crowe MJ, Ying Z, Basso DM (2004) Three exercise paradigms differentially improve sensory recovery after spinal cord contusion in rats. Brain 127: 1403–1414. 15069022 10.1093/brain/awh160

[31] De Mello MT, Esteves AM, Tufik S (2004) Comparison between dopaminergic agents and physical exercise as treatment for periodic limb movements in patients with spinal cord injury. Spinal Cord 42: 218–221. 15060518 10.1038/sj.sc.3101575

[32] Kiser TS, Reese NB, Maresh T, Hearn S, Yates C, et al (2005) Use of a motorized bicycle exercise trainer to normalize frequency-dependent habituation of the H-reflex in spinal cord injury. J Spinal Cord Med 28: 241–245. 16048142 10.1080/10790268.2005.11753818

[33] Phadke CP, Flynn SM, Thompson FJ, Behrman AL, Trimble MH, et al (2009) Comparison of single bout effects of bicycle training versus locomotor training on paired reflex depression of the soleus H-reflex after motor incomplete spinal cord injury. Arch Phys Med Rehabil 90: 1218–1228. 19577036 10.1016/j.apmr.2009.01.022

[34] Rayegani SM, Shojaee H, Sedighipour L, Soroush MR, Baghbani M, et al (2011) The effect of electrical passive cycling on spasticity in war veterans with spinal cord injury. Front Neurol 2: 39. 21734906 10.3389/fneur.2011.00039 PMC3119861

[35] Hangartner TN, Rodgers MM, Glaser RM, Barre PS (1994) Tibial bone density loss in spinal cord injured patients: effects of FES exercise. J Rehabil Res Dev 31: 50–61. 8035360

[36] Lauer RT, Smith BT, Mulcahey MJ, Betz RR, Johnston TE (2011) Effects of cycling and/or electrical stimulation on bone mineral density in children with spinal cord injury. Spinal Cord 49: 917–923. 21423253 10.1038/sc.2011.19

[37] Phillips WT, Kiratli BJ, Sarkarati M, Weraarchakul G, Myers J, et al (1998) Effect of spinal cord injury on the heart and cardiovascular fitness. Curr Probl Cardiol 23: 641–716. 9830574 10.1016/s0146-2806(98)80003-0

[38] Phillips AA, Cote AT, Warburton DE (2011) A systematic review of exercise as a therapeutic intervention to improve arterial function in persons living with spinal cord injury. Spinal Cord 49: 702–714. 21339761 10.1038/sc.2010.193

[39] Faghri PD, Van Meerdervort HF, Glaser RM, Figoni SF (1997) Electrical stimulation-induced contraction to reduce blood stasis during arthroplasty. IEEE Trans Rehabil Eng 5: 62–69. 9086386 10.1109/86.559350

[40] Twist DJ, Culpepper-Morgan JA, Ragnarsson KT, Petrillo CR, Kreek MJ (1992) Neuroendocrine changes during functional electrical stimulation. Am J Phys Med Rehabil 71: 156–163. 1627280 10.1097/00002060-199206000-00006

[41] Seifert T, Secher NH (2011) Sympathetic influence on cerebral blood flow and metabolism during exercise in humans. Prog Neurobiol 95: 406–426. 21963551 10.1016/j.pneurobio.2011.09.008

[42] van Praag H, Christie BR, Sejnowski TJ, Gage FH (1999) Running enhances neurogenesis, learning, and long-term potentiation in mice. Proc Natl Acad Sci U S A 96: 13427–13431. 10557337 10.1073/pnas.96.23.13427 PMC23964

[43] van Praag H, Kempermann G, Gage FH (1999) Running increases cell proliferation and neurogenesis in the adult mouse dentate gyrus. Nat Neurosci 2: 266–270. 10195220 10.1038/6368

[44] Ang ET, Gomez-Pinilla F (2007) Potential therapeutic effects of exercise to the brain. Curr Med Chem 14: 2564–2571. 17979709 10.2174/092986707782023280

[45] Cotman CW, Berchtold NC, Christie LA (2007) Exercise builds brain health: key roles of growth factor cascades and inflammation. Trends Neurosci 30: 464–472. 17765329 10.1016/j.tins.2007.06.011

[46] Endo T, Spenger C, Tominaga T, Brene S, Olson L (2007) Cortical sensory map rearrangement after spinal cord injury: fMRI responses linked to Nogo signalling. Brain 130: 2951–2961. 17913768 10.1093/brain/awm237

[47] Gomez-Pinilla F, Ying Z, Zhuang Y (2012) Brain and spinal cord interaction: protective effects of exercise prior to spinal cord injury. PLoS One 7: e32298. 22384207 10.1371/journal.pone.0032298 PMC3284558

[48] Skinner RD, Houle JD, Reese NB, Berry CL, Garcia-Rill E (1996) Effects of exercise and fetal spinal cord implants on the H-reflex in chronically spinalized adult rats. Brain Res 729: 127–131. 8874885

[49] Viswanath V, Wu Z, Fonck C, Wei Q, Boonplueang R, et al (2000) Transgenic mice neuronally expressing baculoviral p35 are resistant to diverse types of induced apoptosis, including seizure-associated neurodegeneration. Proc Natl Acad Sci U S A 97: 2270–2275. 10681443 10.1073/pnas.030365297 PMC15790

[50] Huang EJ, Reichardt LF (2001) Neurotrophins: roles in neuronal development and function. Annu Rev Neurosci 24: 677–736. 11520916 10.1146/annurev.neuro.24.1.677 PMC2758233

[51] Wang H, Ferguson GD, Pineda VV, Cundiff PE, Storm DR (2004) Overexpression of type-1 adenylyl cyclase in mouse forebrain enhances recognition memory and LTP. Nat Neurosci 7: 635–642. 15133516 10.1038/nn1248

[52] Mi S, Miller RH, Lee X, Scott ML, Shulag-Morskaya S, et al (2005) LINGO-1 negatively regulates myelination by oligodendrocytes. Nat Neurosci 8: 745–751. 15895088 10.1038/nn1460

[53] Ji B, Li M, Wu WT, Yick LW, Lee X, et al (2006) LINGO-1 antagonist promotes functional recovery and axonal sprouting after spinal cord injury. Mol Cell Neurosci 33: 311–320. 17011208 10.1016/j.mcn.2006.08.003

[54] Inoue H, Lin L, Lee X, Shao Z, Mendes S, et al (2007) Inhibition of the leucine-rich repeat protein LINGO-1 enhances survival, structure, and function of dopaminergic neurons in Parkinson’s disease models. Proc Natl Acad Sci U S A 104: 14430–14435. 17726113 10.1073/pnas.0700901104 PMC1955463

[55] Bekinschtein P, Cammarota M, Igaz LM, Bevilaqua LR, Izquierdo I, et al (2007) Persistence of long-term memory storage requires a late protein synthesis- and BDNF- dependent phase in the hippocampus. Neuron 53: 261–277. 17224407 10.1016/j.neuron.2006.11.025

[56] Bekinschtein P, Cammarota M, Katche C, Slipczuk L, Rossato JI, et al (2008) BDNF is essential to promote persistence of long-term memory storage. Proc Natl Acad Sci U S A 105: 2711–2716. 18263738 10.1073/pnas.0711863105 PMC2268201

[57] Mendonca HR, Araujo SE, Gomes AL, Sholl-Franco A, da Cunha Faria Melibeu A, et al (2010) Expression of GAP-43 during development and after monocular enucleation in the rat superior colliculus. Neurosci Lett 477: 23–27. 20406666 10.1016/j.neulet.2010.04.027

[58] Petrinovic MM, Duncan CS, Bourikas D, Weinman O, Montani L, et al (2010) Neuronal Nogo-A regulates neurite fasciculation, branching and extension in the developing nervous system. Development 137: 2539–2550. 20573699 10.1242/dev.048371

[59] Mizutani K, Sonoda S, Yamada K, Beppu H, Shimpo K (2011) Alteration of protein expression profile following voluntary exercise in the perilesional cortex of rats with focal cerebral infarction. Brain Res 1416: 61–68. 21890113 10.1016/j.brainres.2011.08.012

[60] Wei HF, Zeng BF, Chen YF, Chen L, Gu YD (2011) BDNF and GAP43 contribute to dynamic transhemispheric functional reorganization in rat brain after contralateral C7 root transfer following brachial plexus avulsion injuries. Neurosci Lett 500: 187–191. 21723373 10.1016/j.neulet.2011.06.029

[61] Lopes JP, Agostinho P (2011) Cdk5: multitasking between physiological and pathological conditions. Prog Neurobiol 94: 49–63. 21473899 10.1016/j.pneurobio.2011.03.006

[62] Su SC, Tsai LH (2011) Cyclin-dependent kinases in brain development and disease. Annu Rev Cell Dev Biol 27: 465–491. 21740229 10.1146/annurev-cellbio-092910-154023

[63] Fumagalli F, Madaschi L, Caffino L, Marfia G, Di Giulio AM, et al (2009) Acute spinal cord injury reduces brain derived neurotrohic factor expression in rat hippocampus. Neuroscience 159: 936–939. 19344636 10.1016/j.neuroscience.2009.01.030

[64] Felix MS, Popa N, Djelloul M, Boucraut J, Gauthier P, et al (2012) Alteration of forebrain neurogenesis after cervical spinal cord injury in the adult rat. Front Neurosci 6: 45. 22509147 10.3389/fnins.2012.00045 PMC3321502

[65] Zhu HC, Wang LM, Wang M, Song B, Tan S, et al.. (2012) MicroRNA-195 downregulates Alzheimer’s disease amyloid-beta production by targeting BACE1. Brain Res Bull. 10.1016/j.brainresbull.2012.05.018 22721728

[66] Mosyak L, Wood A, Dwyer B, Buddha M, Johnson M, et al (2006) The structure of the Lingo-1 ectodomain, a module implicated in central nervous system repair inhibition. J Biol Chem 281: 36378–36390. 17005555 10.1074/jbc.M607314200

[67] Erzurumlu RS, Jhaveri S, Benowitz LI (1990) Transient patterns of GAP-43 expression during the formation of barrels in the rat somatosensory cortex. J Comp Neurol 292: 443–456. 2160480 10.1002/cne.902920310

[68] Maier DL, Mani S, Donovan SL, Soppet D, Tessarollo L, et al (1999) Disrupted cortical map and absence of cortical barrels in growth-associated protein (GAP)-43 knockout mice. Proc Natl Acad Sci U S A 96: 9397–9402. 10430954 10.1073/pnas.96.16.9397 PMC17794

[69] Gomez-Pinilla F, Ying Z, Roy RR, Molteni R, Edgerton VR (2002) Voluntary exercise induces a BDNF-mediated mechanism that promotes neuroplasticity. J Neurophysiol 88: 2187–2195. 12424260 10.1152/jn.00152.2002

[70] Griesbach GS, Hovda DA, Molteni R, Wu A, Gomez-Pinilla F (2004) Voluntary exercise following traumatic brain injury: brain-derived neurotrophic factor upregulation and recovery of function. Neuroscience 125: 129–139. 15051152 10.1016/j.neuroscience.2004.01.030

[71] Vaynman S, Gomez-Pinilla F (2005) License to run: exercise impacts functional plasticity in the intact and injured central nervous system by using neurotrophins. Neurorehabil Neural Repair 19: 283–295. 16263961 10.1177/1545968305280753

[72] Welker E, Armstrong-James M, Bronchti G, Ourednik W, Gheorghita-Baechler F, et al (1996) Altered sensory processing in the somatosensory cortex of the mouse mutant barrelless. Science 271: 1864–1867. 8596955 10.1126/science.271.5257.1864

[73] Abdel-Majid RM, Leong WL, Schalkwyk LC, Smallman DS, Wong ST, et al (1998) Loss of adenylyl cyclase I activity disrupts patterning of mouse somatosensory cortex. Nat Genet 19: 289–291. 9662407 10.1038/980

[74] Lu HC, She WC, Plas DT, Neumann PE, Janz R, et al (2003) Adenylyl cyclase I regulates AMPA receptor trafficking during mouse cortical ‘barrel’ map development. Nat Neurosci 6: 939–947. 12897788 10.1038/nn1106

[75] Ravary A, Muzerelle A, Herve D, Pascoli V, Ba-Charvet KN, et al (2003) Adenylate cyclase 1 as a key actor in the refinement of retinal projection maps. J Neurosci 23: 2228–2238. 12657682 10.1523/JNEUROSCI.23-06-02228.2003 PMC6742000

[76] Nicol X, Muzerelle A, Rio JP, Metin C, Gaspar P (2006) Requirement of adenylate cyclase 1 for the ephrin-A5-dependent retraction of exuberant retinal axons. J Neurosci 26: 862–872. 16421306 10.1523/JNEUROSCI.3385-05.2006 PMC6675379

[77] Wei F, Qiu CS, Kim SJ, Muglia L, Maas JW, et al (2002) Genetic elimination of behavioral sensitization in mice lacking calmodulin-stimulated adenylyl cyclases. Neuron 36: 713–726. 12441059 10.1016/s0896-6273(02)01019-x

[78] Li XY, Ko HG, Chen T, Descalzi G, Koga K, et al (2010) Alleviating neuropathic pain hypersensitivity by inhibiting PKMzeta in the anterior cingulate cortex. Science 330: 1400–1404. 21127255 10.1126/science.1191792

[79] Jain N, Florence SL, Kaas JH (1995) Limits on plasticity in somatosensory cortex of adult rats: hindlimb cortex is not reactivated after dorsal column section. J Neurophysiol 73: 1537–1546. 7643165 10.1152/jn.1995.73.4.1537

[80] Ghosh A, Haiss F, Sydekum E, Schneider R, Gullo M, et al (2010) Rewiring of hindlimb corticospinal neurons after spinal cord injury. Nat Neurosci 13: 97–104. 20010824 10.1038/nn.2448

[81] Aguilar J, Humanes-Valera D, Alonso-Calvino E, Yague JG, Moxon KA, et al (2010) Spinal cord injury immediately changes the state of the brain. J Neurosci 30: 7528–7537. 20519527 10.1523/JNEUROSCI.0379-10.2010 PMC3842476

[82] Yague JG, Foffani G, Aguilar J (2011) Cortical hyperexcitability in response to preserved spinothalamic inputs immediately after spinal cord hemisection. Exp Neurol 227: 252–263. 21093438 10.1016/j.expneurol.2010.11.011

[83] Moxon KA, Hale LL, Aguilar J, Foffani G (2008) Responses of infragranular neurons in the rat primary somatosensory cortex to forepaw and hindpaw tactile stimuli. Neuroscience 156: 1083–1092. 18775766 10.1016/j.neuroscience.2008.08.009

[84] Darian-Smith C, Gilbert CD (1994) Axonal sprouting accompanies functional reorganization in adult cat striate cortex. Nature 368: 737–740. 8152484 10.1038/368737a0

[85] Yamahachi H, Marik SA, McManus JN, Denk W, Gilbert CD (2009) Rapid axonal sprouting and pruning accompany functional reorganization in primary visual cortex. Neuron 64: 719–729. 20005827 10.1016/j.neuron.2009.11.026 PMC2818836

[86] Marik SA, Yamahachi H, McManus JN, Szabo G, Gilbert CD (2010) Axonal dynamics of excitatory and inhibitory neurons in somatosensory cortex. PLoS Biol 8: e1000395. 20563307 10.1371/journal.pbio.1000395 PMC2885981

[87] Keck T, Scheuss V, Jacobsen RI, Wierenga CJ, Eysel UT, et al (2011) Loss of sensory input causes rapid structural changes of inhibitory neurons in adult mouse visual cortex. Neuron 71: 869–882. 21903080 10.1016/j.neuron.2011.06.034

[88] House DR, Elstrott J, Koh E, Chung J, Feldman DE (2011) Parallel regulation of feedforward inhibition and excitation during whisker map plasticity. Neuron 72: 819–831. 22153377 10.1016/j.neuron.2011.09.008 PMC3240806

[89] Faggin BM, Nguyen KT, Nicolelis MA (1997) Immediate and simultaneous sensory reorganization at cortical and subcortical levels of the somatosensory system. Proc Natl Acad Sci U S A 94: 9428–9433. 9256499 10.1073/pnas.94.17.9428 PMC23207

[90] Jones EG, Pons TP (1998) Thalamic and brainstem contributions to large-scale plasticity of primate somatosensory cortex. Science 282: 1121–1125. 9804550 10.1126/science.282.5391.1121

[91] Jones EG (2000) Cortical and subcortical contributions to activity-dependent plasticity in primate somatosensory cortex. Annu Rev Neurosci 23: 1–37. 10845057 10.1146/annurev.neuro.23.1.1

[92] Jain N, Qi HX, Collins CE, Kaas JH (2008) Large-scale reorganization in the somatosensory cortex and thalamus after sensory loss in macaque monkeys. J Neurosci 28: 11042–11060. 18945912 10.1523/JNEUROSCI.2334-08.2008 PMC2613515

[93] Graziano A, Jones EG (2009) Early withdrawal of axons from higher centers in response to peripheral somatosensory denervation. J Neurosci 29: 3738–3748. 19321770 10.1523/JNEUROSCI.5388-08.2009 PMC2977941

[94] Nothias JM, Mitsui T, Shumsky JS, Fischer I, Antonacci MD, et al (2005) Combined effects of neurotrophin secreting transplants, exercise, and serotonergic drug challenge improve function in spinal rats. Neurorehabil Neural Repair 19: 296–312. 16263962 10.1177/1545968305281209

[95] Paxinos G, Watson C (1986) The rat brain in stereotaxic coordinates. Sydney; Orlando: Academic Press. xxvi, [237] p. p.

[96] Chapin JK, Lin CS (1984) Mapping the body representation in the SI cortex of anesthetized and awake rats. J Comp Neurol 229: 199–213. 6438190 10.1002/cne.902290206

[97] Leergaard TB, Alloway KD, Pham TA, Bolstad I, Hoffer ZS, et al (2004) Three-dimensional topography of corticopontine projections from rat sensorimotor cortex: comparisons with corticostriatal projections reveal diverse integrative organization. J Comp Neurol 478: 306–322. 15368533 10.1002/cne.20289

[98] Friedberg MH, Lee SM, Ebner FF (1999) Modulation of receptive field properties of thalamic somatosensory neurons by the depth of anesthesia. J Neurophysiol 81: 2243–2252. 10322063 10.1152/jn.1999.81.5.2243

[99] Wong-Riley M (1979) Changes in the visual system of monocularly sutured or enucleated cats demonstrable with cytochrome oxidase histochemistry. Brain Res 171: 11–28. 223730 10.1016/0006-8993(79)90728-5

